# GAS1 Promotes Ferroptosis of Liver Cells in Acetaminophen-Induced Acute Liver Failure

**DOI:** 10.7150/ijms.85114

**Published:** 2023-09-25

**Authors:** Jinqiu Tao, Cailin Xue, Xiaodong Wang, Huihui Chen, Qiaoyu Liu, Chunping Jiang, Wenjie Zhang

**Affiliations:** 1 Department of General Surgery, Nanjing Drum Tower Hospital Clinical College of Nanjing Medical University, Nanjing, 210008 Jiangsu Province, China.; 2 Department of Hepatobilliary Surgery, Nanjing Drum Tower Hospital, The Affiliated Hospital of Nanjing University Medical School, Nanjing, 210008 Jiangsu Province, China.; 3 Department of General Surgery, Northern Jiangsu People's Hospital, Yangzhou, 225001 Jiangsu Province, China.; 4 Department of Hepatobilliary Surgery, The first affiliated hospital of Anhui medical university, Hefei, 230022 Anhui Province, China.

**Keywords:** growth arrest-specific gene 1, acute liver failure, acetaminophen, ferroptosis

## Abstract

**Purpose:** Acute liver failure (ALF) is a clinically fatal disease that leads to the rapid loss of normal liver function. Acetaminophen (APAP) is a leading cause of drug-induced ALF. Ferroptosis, defined as iron-dependent cell death associated with lipid peroxide accumulation, has been shown to be strongly associated with APAP-induced liver injury. Growth arrest-specific 1 (GAS1) is a growth arrest-specific gene, which is closely related to the inhibition of cell growth and promotion of apoptosis. However, the functional role and underlying mechanism of GAS1 in APAP-induced ferroptosis remain unknown.

**Methods:** We established liver-specific overexpression of GAS1 (GAS1^AAV8-OE)^ mice and the control (GAS1^AAV8-vector^) mice by tail vein injection of male mice with adeno-associated virus. APAP at 500 mg/kg was intraperitoneally injected into these two groups of mice to induce acute liver failure. The shRNA packaged by the lentivirus inhibits GAS1 gene expression in human hepatoma cell line HepaRG (HepaRG-shNC and HepaRG-shGAS1-2) and primary hepatocytes of mice with liver-specific overexpression of GAS1 were isolated and induced by APAP in vitro to further investigate the regulatory role of GAS1 in APAP-induced acute liver failure.

**Results:** APAP-induced upregulation of ferroptosis, levels of lipid peroxides and reactive oxygen species, and depletion of glutathione were effectively alleviated by the ferroptosis inhibitor, ferrostatin-1, and downregulation of GAS1 expression. GAS1 overexpression promoted ferroptosis-induced lipid peroxide accumulation via p53, inhibiting its downstream target, solute carrier family 7 member 11.

**Conclusion:** Collectively, our findings suggest that GAS1 overexpression plays a key role in aggravating APAP-induced acute liver injury by promoting ferroptosis-induced accumulation of lipid peroxides.

## Introduction

Acute liver failure (ALF) is characterized by the rapid loss of liver function. In >50% of cases, ALF is characterized by liver dysfunction, coagulopathy, encephalopathy, multiorgan failure and death^1^,^2^. ALF is characterized by severe hepatic inflammation that results in apoptosis and fulminant necrosis of the liver. ALF has several causes including, but not limited to, drug toxicity, viruses, toxins, and ischemia^3^. The antipyretic and analgesic drug, paracetamol (APAP, also known as acetaminophen), one of the most commonly used drugs in clinical practice, is also a major cause of acute liver injury and even death^1^,^4^. N-acetylcysteine is widely recognized by the Food and Drug Administration of the United States as a clinical therapeutic drug. It is one of the few drugs that can effectively treat APAP-induced acute liver injury^5^,^6^. Although it is effective in treating APAP-induced acute liver injury in the early stages, it also results in a few undesirable side effects.

APAP overdose-induced acute liver injury occurs via a complex mechanism that has not yet been fully elucidated. Accumulation of APAP leads to the production of toxic metabolites, including N-acetyl-p-benzoquinoneimine (NAPQI)^7^. Excess NAPQI in the body consumes glutathione (GSH) in the cytoplasm and mitochondria, while simultaneously harming the liver cells^8^. In addition, abnormal production of reactive oxygen species (ROS) leads to primary hepatotoxicity, GSH, which is a key antioxidant in liver cells that plays a crucial role in the antioxidant defense system of the body^9^,^10^. Increase in ROS levels results in a reduction in the activity of antioxidant enzymes, such as glutathione peroxidase (GPX), via lipid peroxidation, thereby aggravating acute liver injury caused by APAP accumulation^11^,^12^.

In recent years, ferroptosis, another form of non-apoptotic cell death, has been shown to exhibit distinct morphological and biochemical characteristics^13^. ROS accumulation and iron-driven lipid peroxidation characterize ferroptosis, and cancer, neurological diseases, acute renal failure, and liver injury are associated with dysfunctional ferroptosis^14^,^15^. Recent evidence indicates that ferroptosis may cause various liver diseases, including drug-induced liver damage, viral hepatitis, and ALF^16^,^17^. Ferroptosis can also be caused by the depletion of GSH or inactivation of GPX4 and the accumulation of iron-dependent lipid peroxides^18^-^20^. In ferroptosis, GPX4 is the only metabolic enzyme in the GPX subtype that is capable of regulating the lipid peroxide and ROS levels^21^,^22^. According to current research on ferroptosis, lipid peroxidation resulting from iron overload is the primary cause of ferroptosis^13^,^23^. Activation of intracellular toxic ROS can be enhanced by iron, which acts as a catalyst to promote oxidative stress reactions and aggravate liver injury caused by oxidative stress. According to previous studies, cytosolic iron overload can lead to toxic ROS production via the Fenton reaction, further aggravating APAP-induced oxidative stress^24^. A recent study has identified various regulatory pathways, such as the p53 pathway and factors, such as the cystine/glutamate antiporter (SystemXc^-^) and inhibitory compounds, involved in ferroptosis^25^. These studies suggest that GSH depletion causes APAP-induced liver injury, which may contribute to ALF.

Growth arrest-specific1 (GAS1) has been identified as a growth arrest gene that inhibits cell growth and cell transition from G0 to S phase. Evidence suggests that this region of the chromosome is frequently lost in tumors, particularly in human leukemia, bone marrow malignancies, colorectal carcinomas and bladder cancer^26^-^29^. GAS1 has been reported to selectively express and inhibit DNA replication in quiescent NIH3T3 fibroblasts, suggesting its potential role as a tumor suppressor gene^30^. In B16-F10 melanoma cells with high metastasis, GAS1 shows significantly low expression, which inhibits its metastasis, partly by promoting apoptosis^31^; GAS1 expression has been reported to promote cell differentiation in differentiated hind limb muscle cells^26^,^32^. Overexpression of GAS1 reduces the size, proliferation, and malignancy of liver tumors^33^. Studies have shown that overexpression of GAS1 blocks cell proliferation in a p53-dependent manner and that the transactivation function of p53 is optional for GAS1-induced growth arrest, consequently, other intrinsic non-activated functions of p53 (possibly related to apoptosis regulation) may play a role in GAS1-induced growth arrest^34^,^35^. However, whether GAS1 plays a role in drug-induced liver injury remains unclear.

In this study, we found that ferroptosis is significantly increased by the exposure of liver-specific overexpression of GAS1 (GAS1^AAV8-OE^) mice to APAP, suggesting GAS1 as a critical component of acute liver damage. Therefore, targeting GAS1 may be a novel approach to treat APAP-induced hepatotoxicity.

## Materials and Methods

### Vector preparation

AAV8 vectors were designed to express mouse GAS1 with a thyroxine-binding globulin (TBG) promoter produced by WZ Biosciences, Inc (Jinan, Shandong, China). The carrier was stored between -60 and -80°C. After dilution in sterile phosphate-buffered saline (PBS), the final carrier product was stored at 4 °C or on wet ice.

### Mice

Male C57BL/6J mice were obtained from Gempharmatech Co., Ltd (Nanjing, Jiangsu, China) at 6 weeks-of-age. All mice were raised in specific-pathogen free animal rooms, where constant temperature and humidity were maintained in the animal room and in the standard feed; light was providedin12:12h light/day cycle. The design and operation of all animal experiments were approved by the Laboratory Animal Ethics Committee of Nanjing Drum Tower Hospital Clinical College of Nanjing Medical University. In all studies, the Council for the International Organizations of Medical Sciences' International Guiding Principles for Biomedical Research Involving Animals was followed.

### Clinical samples and cell culture

Tissues used in this study were obtained from the Department of Hepatobiliary Surgery at the drum tower hospital affiliated with the Nanjing University Medical College. During hepatectomy for hepatic hemangiomas, normal liver tissues were obtained, while ALF liver tissues were obtained from patients with ALF receiving liver transplants. The written consent was obtained from all patients and the Ethics Committee of Drum Tower Hospital affiliated with Nanjing University Medical College before the use of patient tissue samples in this study. HepaRG cell line was purchased from the American Type Culture Collection (ATCC). In the culture of the HepaRG cell line, Dulbecco's modified Eagle's medium (DMEM) containing 10% fetal bovine serum (FBS) (Vazyme Company, Nanjing, Jiangsu, China) was used. It was maintained at 37°C and 5% CO_2_.

### Reagents and antibodies

APAP (HY-66005), ferrostatin-1 (HY-100579), RSL (HY-100218A) and erastin (HY-15763) were purchased from MedChemExpress (New Jersey, USA). Anti-α-tubulin (ab7291) antibody was purchased from Abcam (Cambridge, UK), anti-GPX4 (381958) antibody was purchased from Zenbio (California USA). Anti-P53 (2527S) antibody was purchased from Cell Signaling Technology (Massachusetts, USA). Anti-GAS1 (17903-1-AP), anti-solute carrier family 7 member 11 (SLC7A11) (26864-1-AP), anti-mouse IgG-horseradish peroxidase (HRP) (SA00001-1), and anti-rabbit IgG-HRP (SA00001-2) antibodies were purchased from Proteintech (Wuhan, Hubei, China).

### Establishment of an APAP-induced liver injury model

We selected nine-weeks-old male mice with weights similar to those of the model mice. We starved the mice for 16 h before administration of APAP (dissolved in PBS at 60°C and cooled to 37°C before injection). APAP (500 mg/kg) was administered to the mice at four time points (0, 6, 12 and 24 h) after stimulation. After administering anesthesia with isoflurane, eyeball blood was collected in a 1.5-mL centrifuge tube and centrifuged at 3000 rpm for 15 min at 4°C. Cervical vertebrae of the mice were dissected. After disinfection, the abdominal cavity was opened to dissect and separate the livers. Liver tissues (50 mg) were taken and placed in a refrigerator at -80°C for subsequent protein and RNA detection. The remaining liver tissue was fixed with paraformaldehyde and subjected to hematoxylin and eosin (H&E) and immunohistochemical (IHC) staining.

### Quantitative reverse transcription-polymerase chain reaction (RT-qPCR)

Total RNA was extracted and RT-qPCR was performed as previously described^36^. The following primers were used: GAS1 (Homo), forward 5′-TGACCTACTGCGGCAAAGTC-3′ and reverse 5′-CTTGACCGACTCGCAGATGG-3′; GAS1 (Mus), forward 5′-CGGCAAGCTTTTCAACGGG-3′ and reverse 5′-TCTCTTTGACCGATTCGCAG-3′; glyceraldehyde 3-phosphate dehydrogenase (GAPDH) (Homo), forward 5′-GATATTGTTGCCATCAATGAC-3′ and reverse 5′-TTGATTTTGGAGGGATCTCG-3′; GAPDH (Mus), forward 5′-CCCAGCTTAGGTTCATCAGGT-3′ and reverse 5′-GGTCAATGAAGGGGTCGTTG-3′. GAPDH was used as the endogenous control for mRNA expression.

### Lentivirus transfection and stable establishment of interfering GAS1 cell line

Several short hairpin RNA (shRNA) sequences, including a nonsense sequence shNC (5′-GTTCTGGATCGTCAGGT-3′) packaged in lentivirus vectors, shGAS1-1 (5′-GGGCTGTCTATTAGCATATTT-3′) and shGAS1-2 (5′-GGGCTGTCTATTAGCATATTT-3′), were designed and synthesized by GenePharma (Shanghai, China) using Lipofectamine 3000 (Invitrogen, California, USA), according to the manufacturer's instructions.

### H&E staining

For H&E staining, tissues were fixed in paraformaldehyde for 24 h and subjected to gradient dehydration and paraffin embedding. H&E staining was performed on 4 mm-thick paraffin sections prior to sealing.

### IHC and immunofluorescence (IF) staining

After paraffinizing the liver tissues, they were incubated at 37 °C overnight. The sections were then deparaffinized and treated with 3% hydrogen peroxide for 15 min, microwaved and naturally cooled for 40 min. Finally, the samples were incubated with anti-GAS1 antibodies, and fluorescent or biotin-labeled secondary antibodies were added to the liver tissues to observe the protein expression.

### Measurement of mitochondrial membrane potential (MMP) and ROS levels

For ROS quantification, Gas1^AAV8-OE^ and Gas1^AAV8-vector^ primary hepatocytes and HepaRG-shNC and HepaRG-shGAS1-2 cells were treated with 10 mM APAP for 24 h and loaded with MitoTracker Red CMXRos (TRC) and ROS fluorescent probe DHE obtained from KeyGEN BioTECH (Nanjing, Jiangsu, China) and Beyotime Biotech (Shanghai, China), respectively. TRC and ROS levels were determined by measuring the fluorescence excitation/emission spectra at 518/605 and 579/599 nm, respectively, using a laser confocal microscope (Leica, Heidelberg, Germany).

### Liver biochemistry and measurement of MDA and GSH levels

Plasma samples were analyzed using Beckman AU680 (Bria, California, USA) to determine the alanine aminotransferase (ALT), aspartate aminotransferase (AST), creatinine, and lactate dehydrogenase (LDH) levels. We also determined the malondialdehyde (MDA) and GSH levels in HepaRG cells and primary hepatocyte lysates using the MDA assay kit (Beyotime, Shanghai, China) and GSH assay kit (Nanjing Jiancheng, Nanjing, Jiangsu, China), respectively, according to the manufacturer's instructions.

### Primary hepatocyte culture

Primary hepatocytes were isolated from anesthetized mice after perfusion with collagenase solution^37^-^39^. To expose the abdominal vasculature, the intestine was gently displaced using a blunt instrument. The inferior abdominal vena cava (IVC) was located and a suture was placed beneath it, distal to the renal vein. The catheter and needle were placed beyond the suture level of the IVC, distal to the suture. To hold the catheter in place, a suture was tied around it. The needle was then removed carefully. If performed correctly, blood flows through the catheter. The tubing was carefully attached to the catheter. The lung cavity was dissected open to gently reflect the superior liver lobes to expose the diaphragm. Using sharp tip scissors, the diaphragm was punctured and a lateral incision was made to expose the pleural cavity, taking care to avoid the gall bladder and pleural vessels. A bulldog clamp was placed around the inferior thoracic vena cava, proximal to the hepatic vein. Liver was perfused for 5 min with the Liver Perfusion Medium by cutting the portal vein and turning on the pump at a rate of 3-4 mL/min. After 5 min of incubation, the pump was stopped, and the tubing was transferred to the Liver Digest Medium. The pump was later restarted, and the liver was perfused for another 10-15 min. After carefully dissecting the liver, the gallbladder was removed, and the liver was placed in a 10-cm tissue culture dish. Then, 10 mL of the medium was added to the liver in a biosafety cabinet. The liver was gently scraped to remove the hepatocytes. In a 50-mL centrifug tube, the suspension was filtered through a 100-μm cell strainer. The plate was washed with 10mL of cell medium, and the cells were pooled in a conical tube. Cells were centrifuged for 5 min at 350×*g* and 4 °C. The pellet was resuspendedin 10 mL of cell medium, 10% colloidal silica coated with polyvinylpyrrolidone was added, mixed gently, and centrifuged for 5 min at 4 °C. Dead cells appeared at the top of the mixture after centrifugation, while live cells settled at the bottom. The dead cells and medium were aspirated. Cells were washed twice with 20 mL of cell medium, centrifuged for 5 min at 350×*g* and 4 °C, and resuspendedin 10 mL of medium.

### Transmission electron microscopy (TEM)

Left liver lobe was resected, fixed in glutaraldehyde, dehydrated with gradientethanol, and cut into ultrathin slices. The slices were then stained with lead citrate and uranyl acetate and observed under a Hitachi HT-7700 transmission electron microscope (Hitachi. Co. Ltd., Tokyo, Japan).

### Western blot

Liver tissues or cells were extracted using protein extraction kit (Beyotime, Shanghai, China) and protein content was quantified using BCA protein assay kits (Beyotime, Shanghai, China). Protein samples from each group were electrophoresed on a 10-15% sodium dodecyl sulfate-polyacrylamide gel and transferred to polyvinylidene difluoridemembranes. The blots were hatched for 24 h at 4°C with primary antibodies (GAS1, GPX4, SLC7A11, P53 and α-tubulin) after blocking with non-fat dry milk (5%). The blots were washed with tris-buffered saline with tween 20 and incubated with secondary antibodies at 37 °C for 120 min after blocking with non-fat dry milk (5%). An ECL kit was used to detect the bands.

### Statistical analysis

All data are represented as the mean ± standard deviation (mean ± SD). Kaplan-Meier analysis was performed to determine the rate of survival. Student's *t*-test was used to compare the data of two groups. Statistical software (SPSS 23.0) was used to analyze the data and GraphPad Prism version 9.0.0 was used to draw a chart. *P*-values < 0.05 was considered as statistically significant.

## Results

### GAS1 expression is upregulated in ALF

Using real-time PCR, we analyzed GAS1 expression levels in the liver tissues of 18 patients with ALF and 30 controls. The control group consisted of normal liver tissues from patients with benign liver tumors who underwent partial hepatectomy. We found that GAS1 mRNA expression levels were significantly higher in the liver tissues of patients with ALF than in those of the control. We also evaluated the protein levels of GAS1 in seven randomly selected ALF and normal liver tissues using western blot. IHC and IF were also used to determine the expression levels of GAS1 in ALF liver tissues. Expression levels of GAS1 protein were high in ALF liver tissues, but low in normal liver tissues (data not shown).

### Establishment of a mice model with liver-specific ectopic expression of GAS1 using AAV8 with the liver-specific promoter TBG

**Figure 1:**
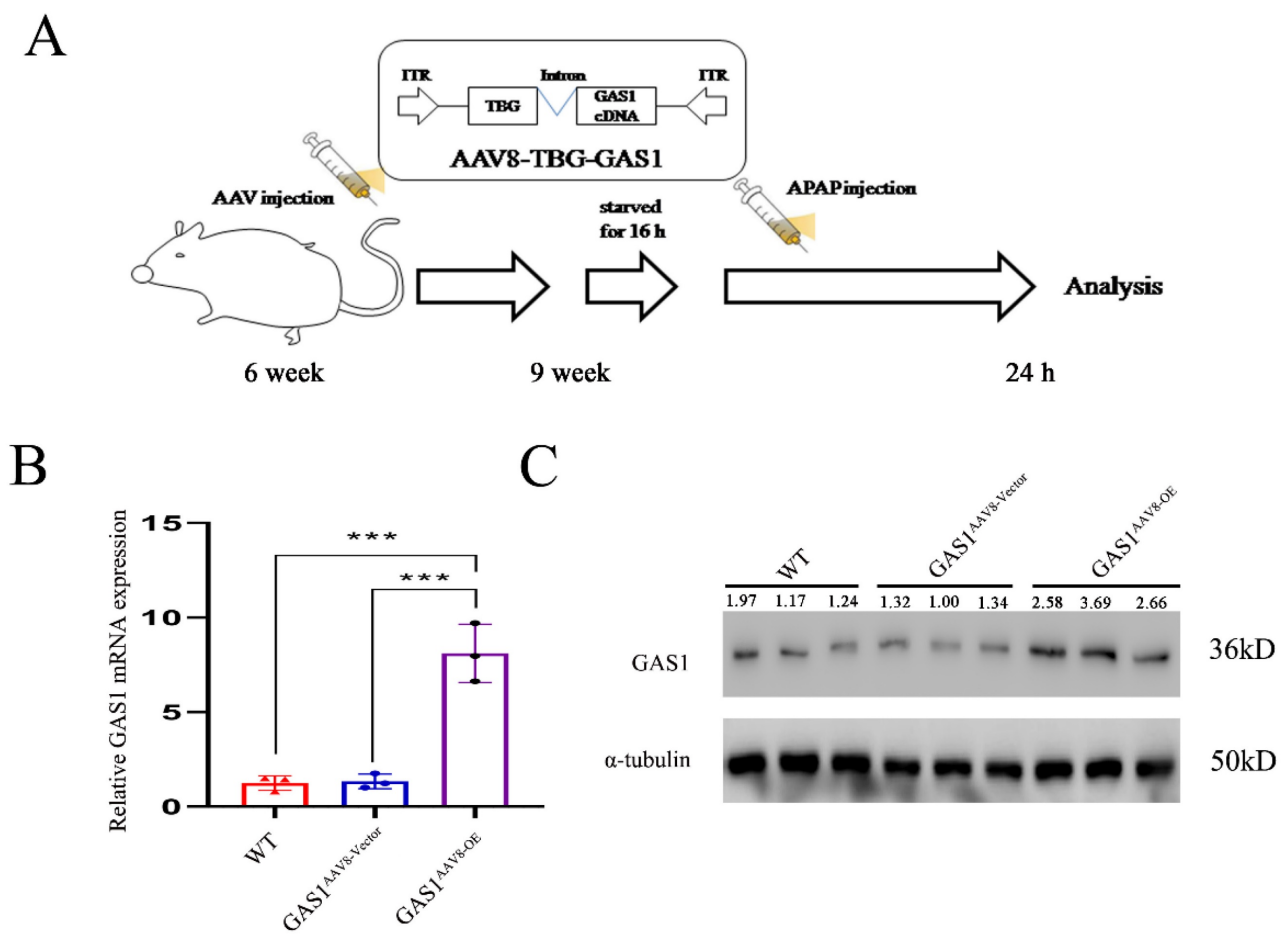
Construction and in vivo liver specific transfection of AAV8-TBG-GAS1. (**A**) Schematic showing the experimental strategy used for GAS1 to aggravate APAP-induced ALF in mice. (**B**) RT-qPCR confirmed the overexpression of GAS1 in the mouse liver. (**C**) Western blot confirmed the overexpression of GAS1 in the mouse liver. Unpaired Student's t-test; *P ≤ 0.05, ***P ≤ 0.001.

GAS1 was ectopically expressed in the liver tissue using AAV8, which is highly efficient in liver transfection, and the liver-specific promoter, TBG^40^. Structural design of theAAV8-TBG-GAS1 carrier and experimental flow chart are shown in Figure 1A. After three weeks, we divided 9-week-old male C57BL/6J mice into three groups, namely the wild-type (WT), vector and GAS1 overexpression groups, to study the effects of GAS1 on APAP-induced liver injury in mice. After APAP treatment, RT-qPCR analysis of revealed an increase in GAS1 mRNA levels in the livers of mice overexpressing GAS1 compared to those in WT and vector mice (Figure 1B). We then conducted western blot to determine the protein expression levels of GAS1 in the three groups and found that the results were consistent with the RT-qPCR results (Figure 1C).

### Liver-specific ectopic expression of GAS1 aggravates tissue damage after ALF in mice

**Figure 2:**
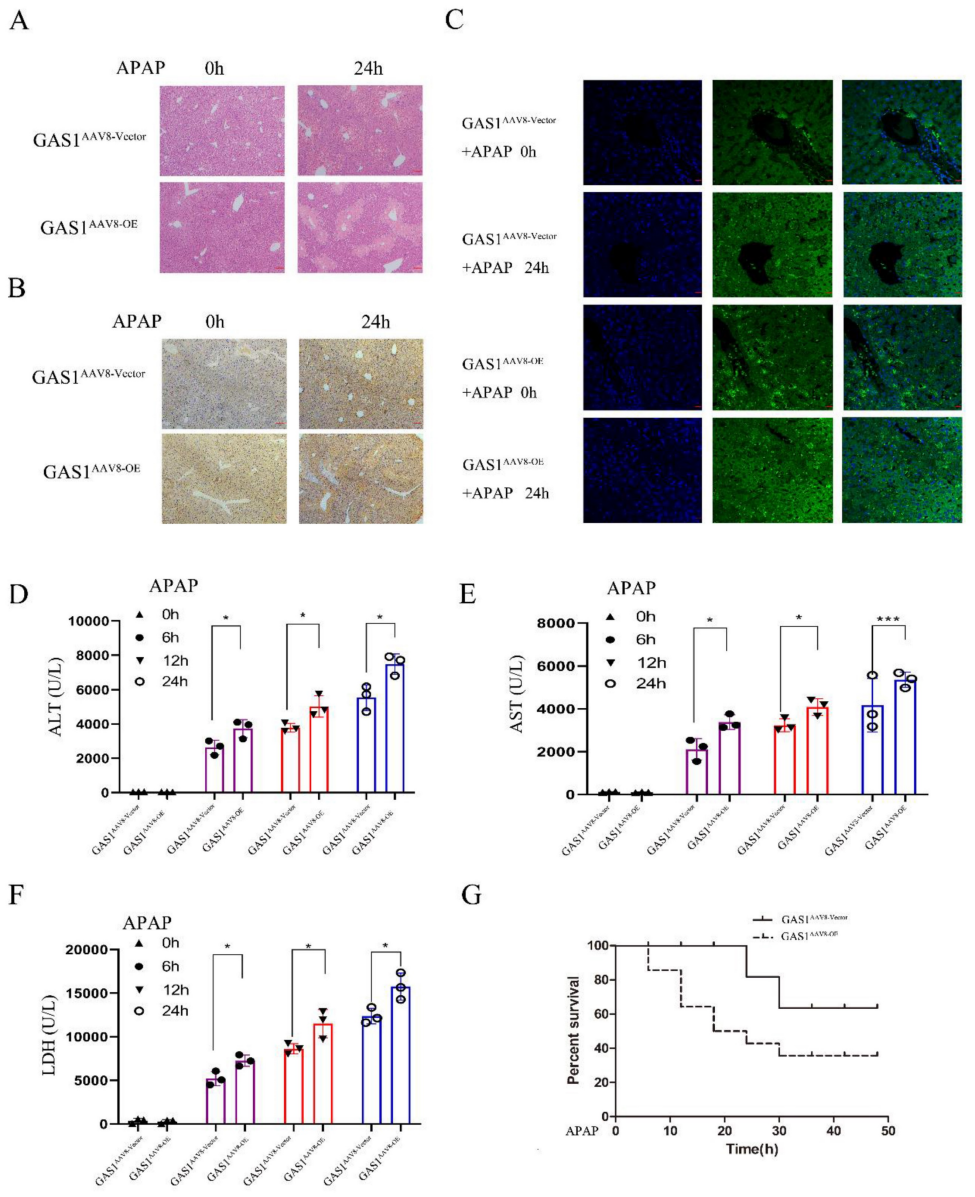
Overexpression of GAS1 aggravated the hepatotoxicity induced by APAP (500mg/kg, 24h) in mice. (**A**) Liver tissues were harvested 24 h after application of APAP and processed for hematoxylin and eosin (H&E) staining; (**B-C**) Immunohistochemical and immunofluorescence staining of GAS1 in the liver tissues of mice treated with APAP for 24 h; (**D-F**) Levels of the serum markers, AST, ALT and LDH were measured; (**G**) Survival curve of GAS1 vector and OE mice treated with a dose of APAP. All mice were observed for survival 48 h after APAP treatment. Unpaired Student's t-test; *P ≤ 0.05, ***P ≤ 0.001.

After 24 h of APAP treatment, H&E staining revealed that the degree of damage in the GAS1 overexpression group was significantly higher than that in the carrier group, with edema and necrosis of hepatocytes observed around the central vein of the hepatic lobule (Figure 2A). Meanwhile, GAS1 immunohistochemical expression levels in the livers of the overexpression group were significantly higher than those in the overexpression group without APAP treatment (Figure 2B). Moreover, IHC and IF results in the liver tissues also demonstrated that mice overexpressing GAS1 in hepatocytes had significantly higher levels of GAS1 staining in their livers compared with 0h, indicating that the expression of GAS1 protein is significantly higher after ALF than that without ALF (Figure 2B and 2C). Levels of serum biochemical indicators were also determined (Figures 2D, 2E and 2F). Levels of ALT, AST and LDH increased significantly after APAP treatment. Levels of the biochemical indicators, ALT, AST and LDH were significantly different between the liver-specific overexpression GAS1 and control groups. Additionally, we determined the 48h survival rate of mice in the control and GAS1 overexpression groups following liver injury induced by 500 mg/kg APAP. We found that only 35.71% of mice in the GAS1 overexpression group survived compared to 63.64% of mice that survived in the control group (Figure 2G). GSH of GAS1^AAV8-OE^ and GAS1^AAV8-vector^ mice liver after 1 hour of APAP administration were assessed (Figure S1).

### Knockdown of GAS1 inhibits APAP-induced ferroptosis in HepaRG

**Figure 3:**
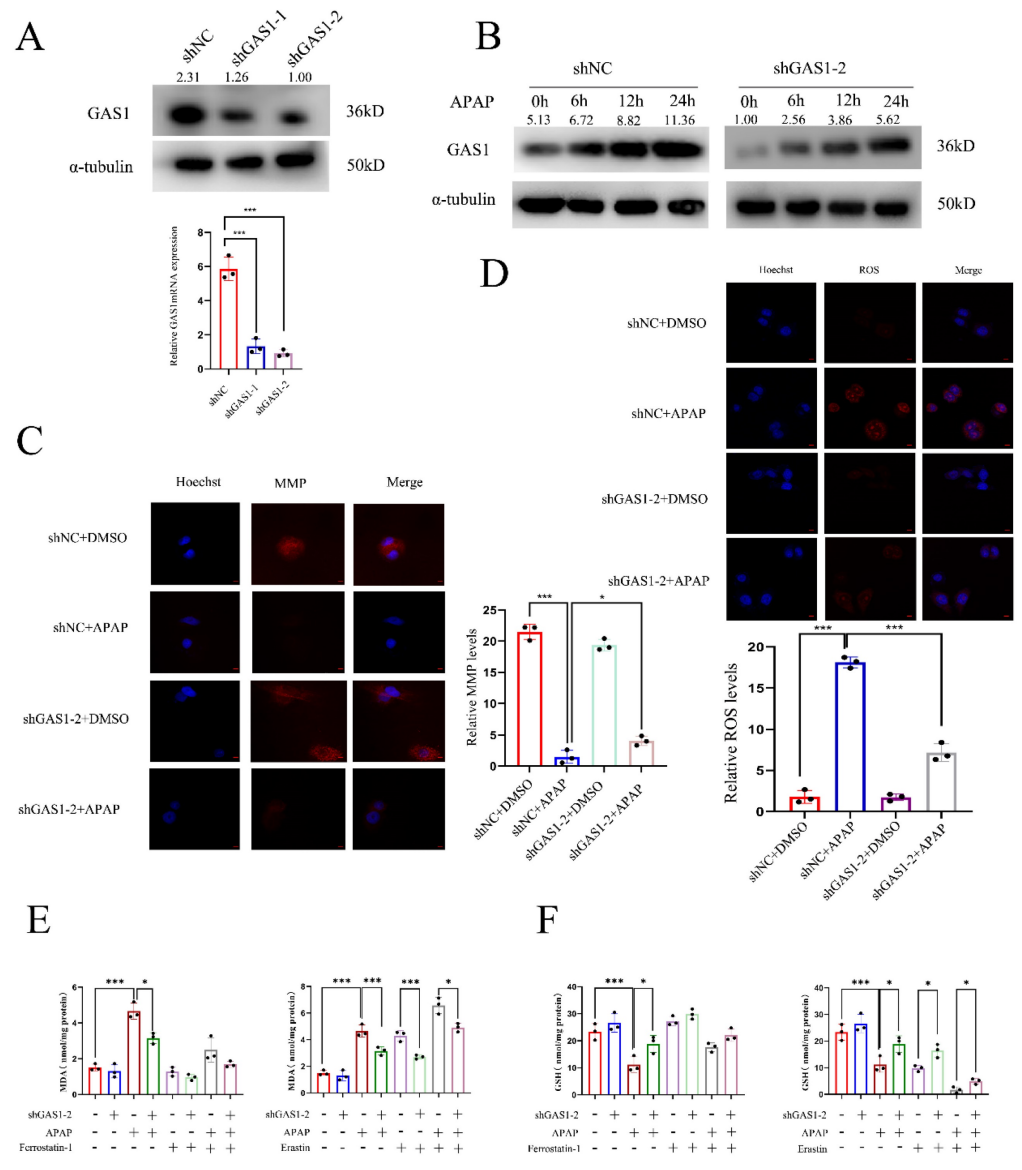
Knockdown of GAS1 alleviates APAP-induced ALF by reducing ferroptosis in HepaRG cells. (**A**) HepaRG cells stably inhibited by shGAS1-2 were examined via western blotting and RT-PCR. (**B**) Expression levels of GAS1 in liver tissues at a specific time point after APAP treatment of HepaRG cells determined via western blotting. (**C-D**) For HepaRG-shNC and HepaRG-shGAS1-2, mitochondrial membrane potential (MMP) and ROS levels were measured 24h after ALF was induced by APAP. (**E-F**) After treatment with ferrostatin-1(1μM) and/or erastin (10μM) for 24 h, malondialdehyde (MDA) and glutathione (GSH) levels in HepaRG-shNC and HepaRG-shGAS1-2 cells were determined in the presence or absence of APAP. Unpaired Student's t-test; *P ≤ 0.05, ***P ≤ 0.001.

In vitro studies of APAP hepatotoxicity commonly use 10 mM APAP as the median lethal dose for hepatocytes^41^,^42^. We established an in vitro APAP model using HepaRG cells. RT-qPCR and western blot analysis were conducted using the constructed stably transfected cell line knocked down by GAS1 (Figure 3A). For subsequent experiments, we selected the stable cell line with the highest interference efficiency. After APAP treatment, GAS1 expression was significantly increased in HepaRG-shGAS1-2 and HepaRG-shNC cells in a time-dependent manner, and indicated that the expression of GAS1 protein is significantly higher after ALF than that without ALF (Figure 3B). Additionally, to verify whether interference with GAS1 expression can inhibit APAP-induced oxidative stress and MMP changes in hepatocytes, we tested the levels of corresponding indicators. TRC and DHE staining results showed that after 24h of APAP treatment, the decrease in MMP in the GAS1 knockdown group was significantly lower than that in the control group (Figure 3C), while ROS levels were reduced in the HepaRG-shGAS1-2 group compared to those the control group (Figure 3D). Ferroptosis is a type of regulated cell death caused by the accumulation of redox-active iron, depletion of GSH and lipid oxidation^43^,^44^. To determine whether GAS1 was involved in the regulation of APAP caused ferroptosis in HepaRG cells, we evaluated the effects of ferroptosis inducers (erastin 10μM) and inhibitors (ferrostatin-1 1μM) on these cells. We found that APAP decreased GSH and increased MDA levels in HepaRG cells, whereas treatment with ferrostatin-1 had the opposite effect and no significant difference between MDA and GSH levels in HepaRG-shGAS1-2 and HepaRG-shNC cells. These results indicate that APAP stimulates HepaRG cells to activate ferroptosis (Figure 3E and 3F). These results indicate that in vitro inhibition of GAS1 inhibits ROS accumulation, alleviates oxidative damage and inhibits ferroptosis caused by APAP.

### Overexpression of GAS1 aggravates APAP-induced ferroptosis of primary mice hepatocytes

**Figure 4:**
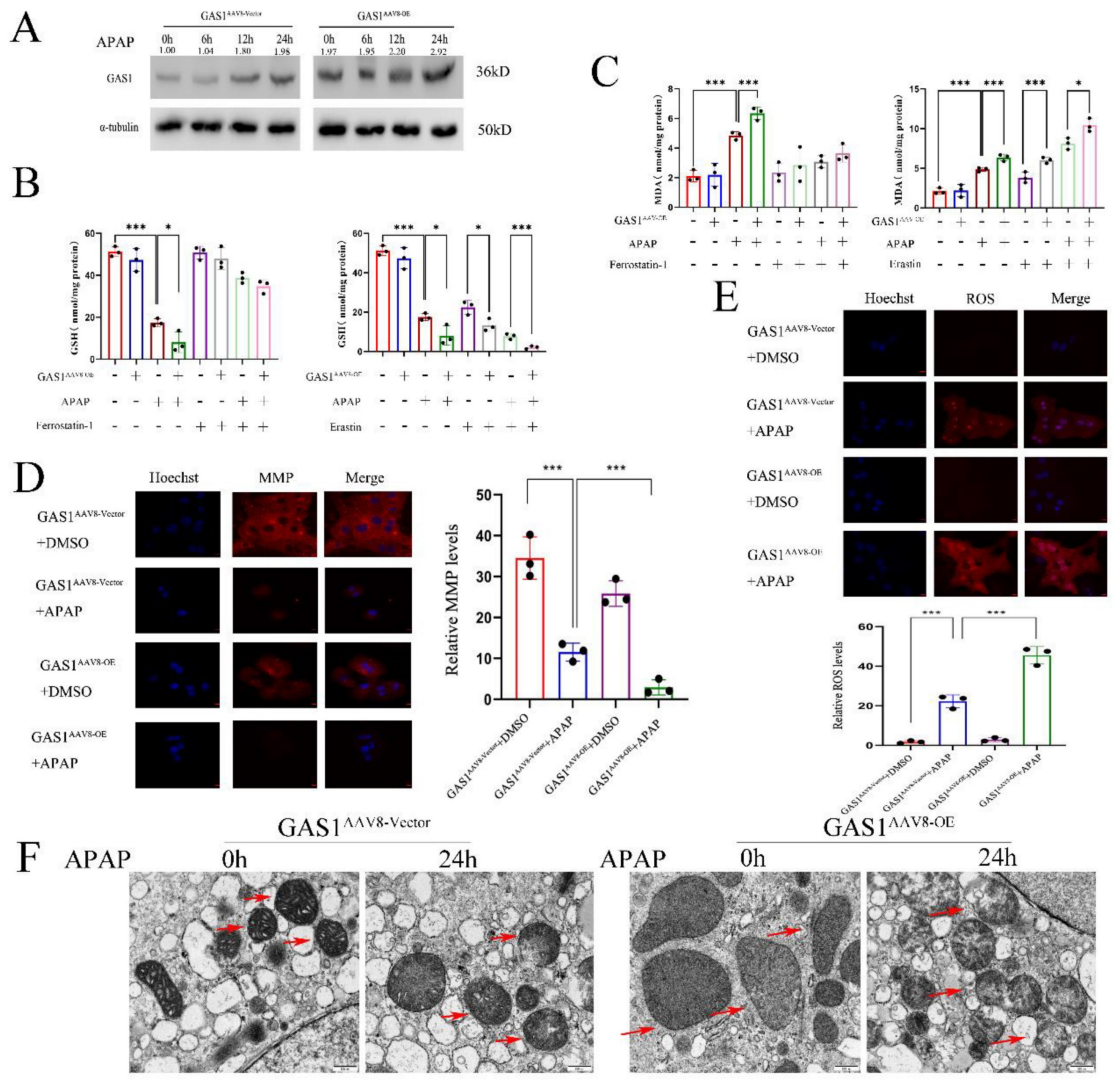
Overexpression of GAS1 in mice primary hepatocytes aggravates APAP-induced ALF by promoting ferroptosis. (**A**) Expression levels of GAS1 in the liver tissues at a specific time point after APAP treatment of mice primary hepatocytes determined via western blotting. (**B-C**) After treatment with ferrostatin-1(1μM) and/or erastin (10 μM) for 24 h, malondialdehyde (MDA) and glutathione (GSH) levels in GAS1^AAV8-Vector^ and GAS1^AAV8-OE^ mice primary hepatocytes were determined in the presence or absence of APAP. (**D-E**) In GAS1^AAV8-Vector^ and GAS1^AAV8-OE^ mice primary hepatocytes, MMP and ROS levels were measured 24h after ALF was induced by APAP. (**F**) Representative images showed by TEM. The red arrow indicates representative mitochondria in GAS1^AAV8-Vector^ and GAS1^AAV8-OE^ mice primary hepatocytes treated by APAP or not. Unpaired Student's t-test; *P ≤ 0.05, ***P ≤ 0.001.

To further assess the effects of GAS1 on APAP-induced liver injury, we induced ferroptosis in primary mouse hepatocytes. We isolated primary mouse hepatocytes from mice with liver-specific overexpression of GAS1 as well as from those in the vector group. After APAP treatment, GAS1 expression was significantly increased in the GAS1^AAV8-Vector^ and GAS1^AAV-OE^ groups in a time-dependent manner, and indicated that the expression of GAS1 protein is significantly higher after ALF than that without ALF (Figure 4A). APAP treatment of GAS1^AAV8-Vector^ and GAS1^AAV-OE^ groups worked as expected, with GAS1^AAV-OE^ producing more MDA and consuming more GSH than GAS1^AAV8-Vector^. Erastin (10μM) and ferrostatin-1 (1μM) were evaluated for the participation of GAS1 in primary hepatocyte ferroptosis. Erastin decreased the levels of GSH and increased the levels of MDA induced by APAP in GAS1^AAV8-OE^ mice, whereas ferrostatin-1 increased the levels of GSH and MDA (Figure 4B and 4C). Similarly, we treated GAS1^AAAV8-Vector^ and GAS1^AAAV-OE^ primary mouse hepatocytes with APAP. MMP and ROS levels were measured after 24h. MMP was decreased in GAS1^AAV8-OE^ cells compared to that in GAS1^ AAV8-Vector^ cells, while ROS levels were increased in GAS1^AAAV-OE^ cells compared to that in GAS1^AAV8-Vector^ (Figure 4D and 4E). Ferroptosis is distinguishable by morphological changes in mitochondria. The morphology of mitochondria was captured using TEM to determine the root cause of aggravated liver injury. We found that APAP caused significant mitochondrial abnormalities, including the reduction or even disappearance of mitochondrial cristae or rupture of the mitochondrial outer membranes. Overexpression of GAS1 further aggravated mitochondrial damage induced by APAP via ferroptosis (Figure 4F).

### GAS1 promotes APAP-induced hepatotoxicity via the p53/SLC7A11 signaling pathway

**Figure 5:**
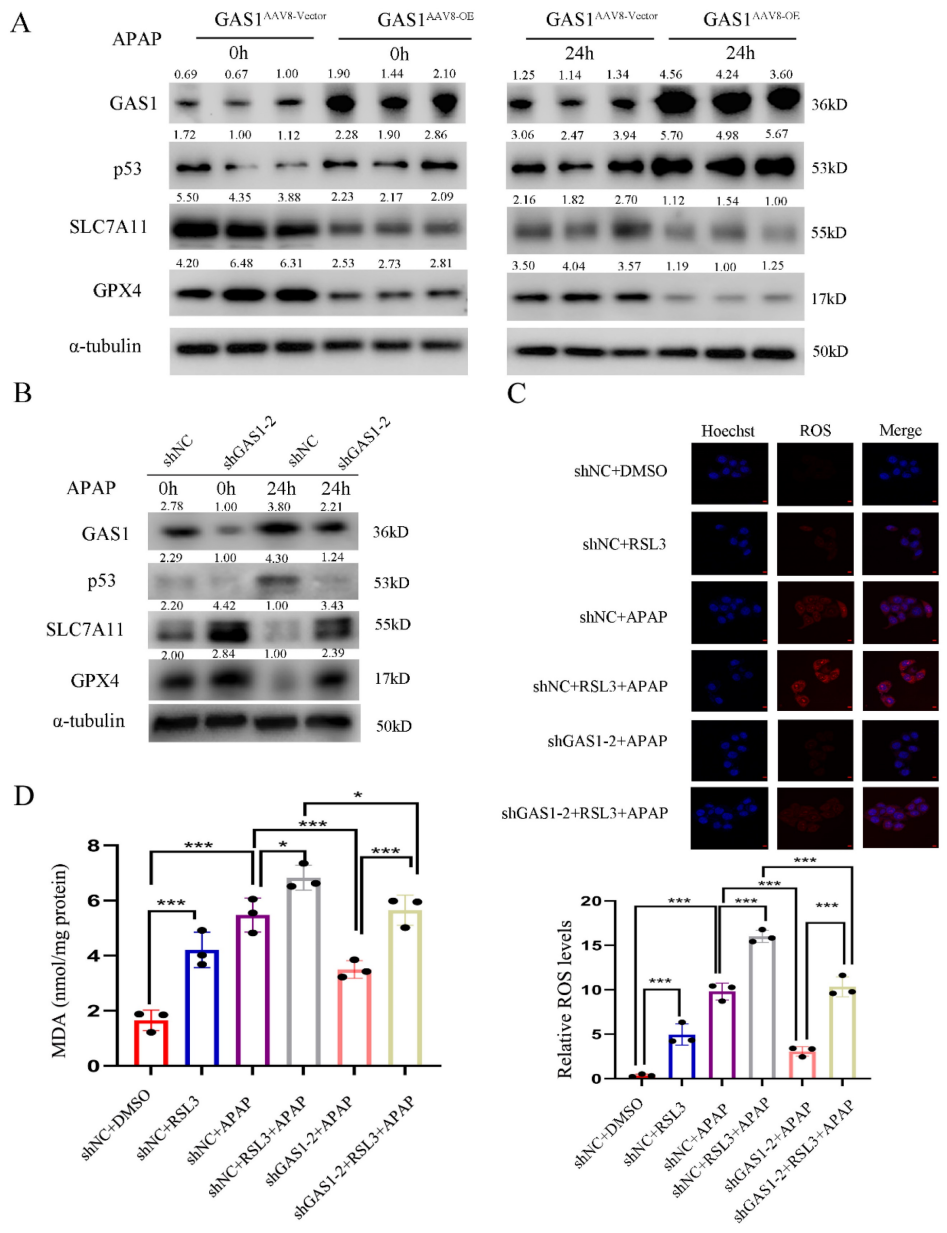
Activation of the p53/SLC7A11/GPX4 pathway is critical for GAS1 to affect ferroptosis in APAP-induced ALF. (**A**) Expression levels of p53, SLC7A11 and GPX4 in GAS1^AAV8-Vector^ and GAS1^AAV8-OE^ mice primary hepatocytes induced by APAP were determined using western blot. (**B**) Western blot analysis of ferroptosis signaling pathway-related proteins, p53, SLC7A11 and GPX4 after transfection of HepaRG cells with shGAS1-2 induced by APAP. (**C-D**) After treatment with RSL3 (1μM) for 24 h, ROS and MDA levels in HepaRG-shNC and HepaRG-shGAS1-2 cells were determined in the presence or absence of APAP. Unpaired Student's t-test; *P ≤ 0.05, ***P ≤ 0.001.

Ferroptosis is a new pathway of cell death that is characterized by an increase in Fe^2+^ levels and MDA, accumulation of ROS and reduction in the synthesis of GSH, which results in the damage of the mitochondrial cristae or rupture of the outer membrane of mitochondria, ultimately leading to cell death^13^. SLC7A11, a part of System Xc^-^ is involved in the production of GSH; therefore, inhibiting its expression can reduce GSH activity. p53 has been reported to inhibit SLC7A11 expression to promote ferroptosis^45^,^46^. Following our demonstration of the role of GAS1 in APAP-induced liver injury, we isolated primary mouse hepatocytes and explored the molecular signaling mechanisms of GAS1 involved in ferroptosis promotion in primary mouse hepatocytes and HepaRG cells. As a result of exposure to APAP, p53 expression levels were significantly higher, but SLC7A11 and GPX4 expression levels were significantly lower in GAS1^AAV8-OE^ primary mouse hepatocytes than in GAS1^AAV8-Vector^ primary mouse hepatocytes (Figure 5A). Inhibition of GAS1 expression in HepaRG cells further confirmed this result (Figure 5B). RSL3 (1μM) significantly increased ROS and MDA production in HepaRG-shNC and HepaRG-shGAS1-2 cells, suggesting that GAS1 may regulate ferroptosis through GPX4 (Figure 5C and 5D). These results suggest that GAS1 aggravates hepatocyte injury caused by APAP by promoting ferroptosis.

## Discussion

ALF is a severe condition. Globally, APAP overdose is the leading cause of acute liver damage and even liver failure, with increasing prevalence in China^2^,^4^. However, relevant treatment methods for it are still limited and safer and more effective treatment strategies urgently needed. Researchers have found that APAP-induced liver oxidative stress and mitochondrial dysfunction are responsible for APAP-induced liver injury caused by oxidative stress and mitochondrial dysfunction^47^,^48^. APAP is believed to cause liver injury as follows: excessive intake of APAP is saturated by liver glucuronidation and sulphuration, and APAP is metabolized to form toxic NAPQI primarily by CYP4502E1, resulting in its degradation by GSH in the liver^49^, eventually causing various forms of liver cell death. Therefore, it is necessary to determine the underlying mechanism of cell death to aid in the clinical treatment of acute liver injury and liver failure caused by excessive APAP.

Recently, ferroptosis has been identified as a new form of non-apoptotic cell death induced by iron-dependent lipid peroxide accumulation, and it is distinct from other forms of cell death based on its morphological and biochemical characteristics^13^. In addition to causing oxidative stress, fibrosis can also result in liver injury as a result of acute liver inflammation and injury 18,50. An experimental study found that ferroptosis can be induced by depletion of GSH, inactivation of GPX4, and accumulation of iron-dependent lipid peroxide^19^,^51^. Ferroptosis is mediated by the GPX4 metabolizing enzyme, which is the only GPX subtype enzyme capable of regulating the lipid peroxide and ROS levels^12^,^52^. Lipid peroxidation caused by iron overload is the primary cause of fibrosis. Iron is a catalyst for intracellular toxic ROS, which promote oxidative stress reactions and enhance liver injury caused by oxidative stress. Through the Fenton reaction, cytosolic iron overload can result in toxic ROS production, which may exacerbate the oxidative stress-induced damage caused by APAP^12^,^53^. The metallothionein, SLC7A11, is part of System Xc^-^; therefore, inhibiting its expression can reduce the GSH activity. p53 has been reported to inhibit SLC7A11 expression to promote ferroptosis^25^,^45^,^54^,^55^.

Studies have shown that GAS1 expression is generally downregulated in many tumor tissues, suggesting its potential function as a tumor suppressor^26^,^27^,^30^. GAS1 can inhibit the proliferation of malignant tumor cells and promote apoptosis in cancers of the rectal wall and melanoma^29^,^31^. In this study, we confirmed that GAS1 expression levels were significantly higher in ALF liver tissues than in normal liver tissues. We established a mouse liver failure model based on APAP-induced liver injury. The results showed that GAS1^AAV8-OE^ mice showed significantly worse in liver injury and inflammation than the control GAS1^AAV8-vector^ mice. Several indices of mouse hepatocytes and HepaRG cells were analyzed before and after exposure of GAS1^AAV8-OE^ and GAS1^AAV8-Vector^ mice and HepaRG-shNC and HepaRG-shGAS1-2 cells to APAP. Use of ferroptosis inhibitor, ferrostatin-1, and downregulation of GAS1 effectively alleviated the APAP-induced upregulation of lipid peroxide and ROS levels and depletion of GSH. Therefore, inhibition of GAS1 may reduce the accumulation of ROS in the liver, thereby reducing liver lipid peroxide levels and further inhibiting ferroptosis and liver injury. The objective of this study was to determine the pathway through which GAS1 plays a role in ferroptosis and which pathway GAS1 acts as the main regulatory pathway of ferroptosis, including antioxidant, iron metabolism, and lipid metabolism pathways. Studies have shown that the overexpression of GAS1 blocks cell proliferation in a p53-dependent manner and that the trans-activation function of p53 is optional for GAS1-induced growth arrest. Therefore, other intrinsic non-activated functions of p53 (possibly related to apoptosis regulation) may play a role in GAS1-induced growth arrest^34^. p53 functions as a tumor suppressor gene. It regulates the cell growth cycle, affects the metabolism of tumor cells, induces apoptosis, and promotes DNA damage^56^. p53 inhibits the activity of System Xc^-^ and the entry of cystine into cells, reduces the synthesis of GSH and decreases the activity of GPX4 after acetylation, thereby promoting ferroptosis^57^,^58^. Recent studies have shown that p53 increases glutaminase 2 activity to catalyze the production of glutamic acid. Because cystine and glutamic acid are transported in a 1:1 ratio, the high concentration of glutamic acid in cells inevitably reduces the entry of cystine into the cells, reducing ferroptosis resulting from GSH synthesis^57^. These studies outline the significance of the relationship between GAS1 and ferroptosis in APAP-induced liver injury mediated by p53. Therefore, we hypothesized that the knockdown or overexpression of GAS1 could attenuate or aggravate APAP-induced ALF via the p53/SLC7A11 signaling pathway. Western blot was used to detect the expression levels of p53, SLC7A11, and GPX4 in GAS1^AAV8-OE^ and GAS1^AAV8-Vector^ primary mouse hepatocytes and HepaRG-shNC and HepaRG-shGAS1-2 cells. Indeed, p53 expression was positively correlated with GAS1 expression, whereas SLC7A11 and GPX4 expression levels were negatively correlated with GAS1 expression.

As many others, this study has some shortcomings. More than one type of hepatocyte death is involved in acute and chronic liver injury, including apoptosis, necroptosis, ferroptosis, pyroptosis and autophagy^59^,^60^. The tandem relationship between hepatocyte death and many other types of cell death in ALF shows that not only ferroptosis plays an important role in acute liver injury, but also other types of hepatocyte death, which provides a direction for our follow-up research. Ferroptosis, a new type of cell death caused by the accumulation of iron-dependent lipid peroxides, has been associated with ALF^61^. Studies have shown that GPX4 regulates the regulatory cell death, including apoptosis, necrosis and autophagy. Previous studies have indicated that the knockout of receptor-interacting protein kinase 1 (RIPK1) or RIPK3 increases the susceptibility of mouse cells to ferroptosis, while inhibition of GPX4 facilitates the induction of apoptosis by apoptosis activators. Recent studies have also demonstrated the relationship between ferroptosis and apoptosis, although further in-depth studies are required to confirm this relationship^62^. GPX4 is an important component of ferroptosis. Overexpression of GPX4 has been reported to inhibit ROS-mediated autophagy and death of immune cells owing to its enhanced antioxidant capacity^63^. Therefore, ALF is characterized not only by single hepatocyte death but also ferroptosis, a relatively new mode of cell death identified in recent years. Although only a few studies have focused on ALF, the tandem relationship between ALF and many other types of cell death suggests that it may play an important role in acute liver injury.

The present study has several limitations. Firstly, the clinical samples we selected included ALF caused by prescription drugs, Chinese herbs, viruses, etc., and APAP-induced liver failure was only an experimental model we used. APAP hepatotoxicity mouse model can well reproduce human pathophysiology, so this clinically relevant model is often used in experimental studies. However, no model can fully restore all the characteristics of human acute liver injury. Different modeling methods involve different experimental procedures, and each animal model has its own advantages, but at the same time there are certain shortcomings. In spite of these limitations, we also found a high expression of GAS1 in the APAP-induced liver failure mouse model, suggesting a need to add additional liver failure models for more detailed study. Based on the clinical data, we presented subsequent hypotheses, which represent a direction for follow-up research. Secondly, our study contains a small sample size and there may be type I or type II statistical errors. The article does not mention the effects of ferroptosis inducers and inhibitors at different doses. Our previous pre-experiments evaluated the effects of different doses of ferroptosis inhibitors and inducers, but were not reported in the article. We selected a dose with the highest effect as the follow-up experiment^64^. We will, if necessary, increase the number of samples and concentration gradient for future validation. At last, the mechanism of APAP-induced ALF is still unclear, and there are different voices in the academic community. The role of lipid peroxidation in APAP-induced ALF has been debated for decades. Some scholars believe that APAP-induced acute liver failure is due to the nitrification of proteins^65^, this view has been supported by evidence showing that, while tocopherol supplementation can reduce APAP-induced lipid peroxidation (LPO), it does not attenuate APAP-induced liver injury, possibly due to its inability to affect peroxynitrite formation^66^. Another study found that a diet deficient in vitamin E and high in soybean oil increased hepatic polyunsaturated fatty acid levels, and this could be the primary pathway of APAP hepatotoxicity. However, in mice, the increase of LPO was very limited in the APAP-induced liver injury, and the amount was insufficient to cause cell death^65^,^67^. In addition, MnSOD catalyzes the distortion of superoxide in mitochondria and prevents the formation of peroxynitrite, MnSOD also produces hydrogen peroxide. Depending on the redox balance, it can be detoxified by glutathione peroxidase or catalase68. It has been found that MnSOD deficiency substantially exacerbates APAP-induced protein nitrification and liver injury^69^. On the contrary, some scholars believe that there is ferroptosis in the process of APAP-induced ALF^12^,^17^,^70^,^71^. In previous studies, ferroptosis has been demonstrated to contribute to APAP-induced ALF in mouse models, is prevented by the ferroptosis-specific inhibitor ferrostatin-1, the iron chelator deferoxamine (DFO) and α-tocopherol and APAP-induced liver injury, LPO and GSH depletion have been reduced by ferroptosis inhibitors. These results suggest that GSH depletion-driven iron depletion may be a major mechanism of APAP-induced toxicity. Mass spectrometry and genetic inhibition of ACSL4 demonstrate that free radical oxidation of n-6 polyunsaturated fatty acids causes APAP-driven hepatocyte ferroptosis^17^. There have been numerous studies examining the role of ferrostatin-1, a specific ferroptosis inhibitor, in APAP-induced cell death in vitro. Ferrostatin-1 is a free radical scavenger and has been demonstrated to protect liver cells from the cellular damage caused by APAP^72^. As demonstrated by Schnellmann et al., DFO inhibits APAP-induced liver injury in a dose-dependent manner. Ferrostatin-1 prevented APAP-induced cell death both in vitro and in vivo. Although ferrostatin-1 had no effect on CYP2E1 expression, pretreatment with this inhibitor resulted in increased GSH levels three hours following APAP treatment.^17^,^72^. According to RNA-seq data and qPCR validation in another group of mice, MnSOD mRNA levels in mouse livers were not upregulated after APAP challenge. However, whether SOD-mediated endogenous defenses are toxic is unknown^73^. In our study, a strong occurrence of both cell death and lipid peroxidation was observed in the livers of APAP-treated mice, accompanied by decreased GPX4 expression and decreased GSH content. The ferroptosis inhibitor ferrostatin-1 greatly attenuated the above changes caused by APAP. This evidence may support that ferroptosis is involved in the pathogenesis of ALF. In addition, our findings show that inhibiting the expression of GAS1 had significant protective and therapeutic effects on APAP-induced ALF. However, the mechanism of APAP-induced ALF is unclear. More research is needed to clarify this in the future.

## Conclusion

**Figure 6:**
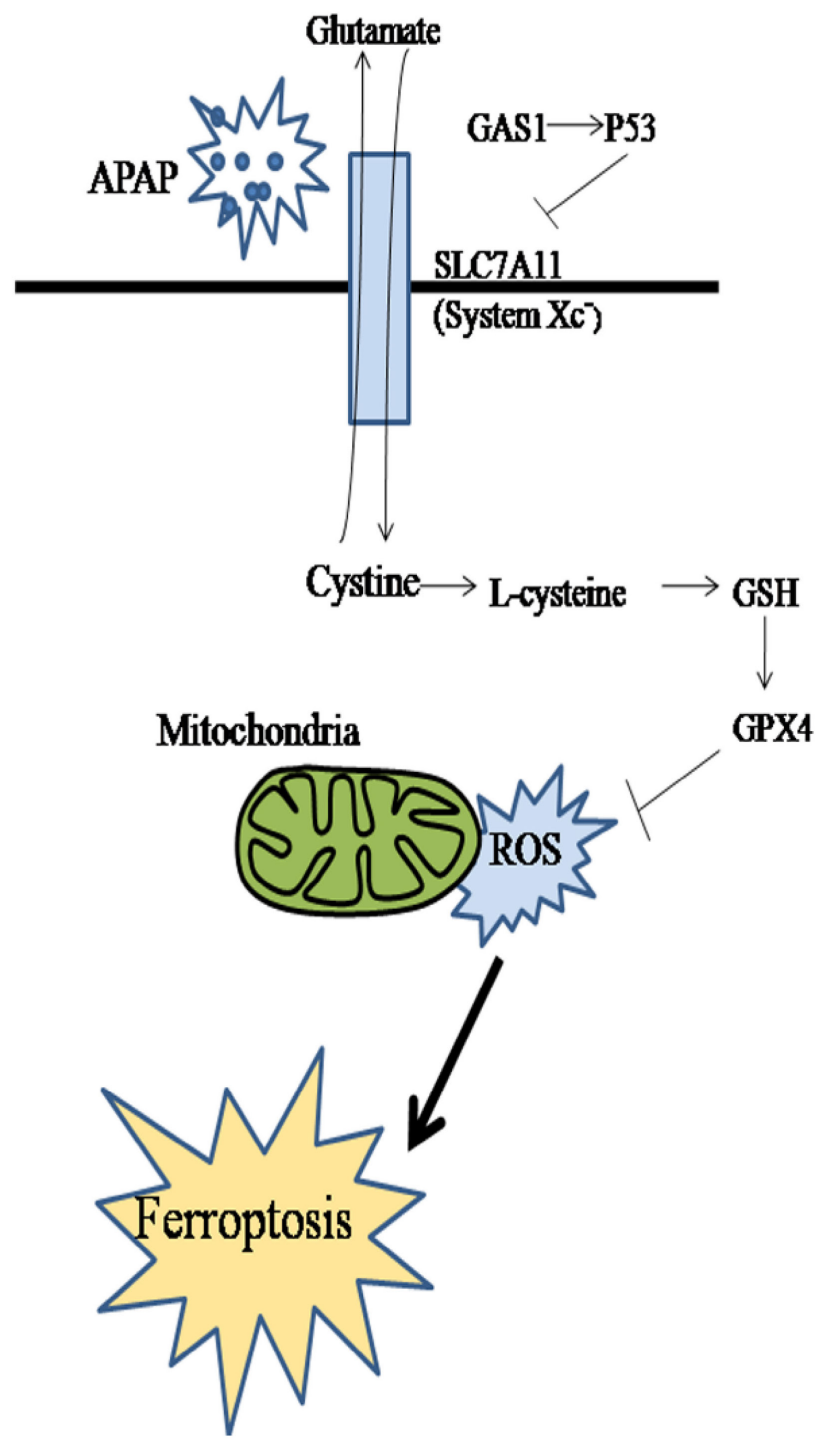
Schematic diagram of the effect and underlying mechanism of GAS1 on ferroptosis in APAP-induced ALF.

In conclusion, we studied the effect of GAS1 on ferroptosis in APAP-induced ALF and found that lipid peroxidation and redox imbalance play key roles in this process. Inhibiting the expression of GAS1 had significant protective and therapeutic effects on APAP-induced ALF (Figure 6). Therefore, ferroptosis may be an effective target for the treatment of APAP-induced ALF. Moreover, GAS1 may be a potential target for ferroptosis that can be used in the clinical treatment of ALF in the future.

## Supplementary Material

Supplementary figure.

## Abbreviations

ALF: acute liver failure
APAP: acetaminophen
GAS1: growth arrest-specific1
GPX4: glutathione peroxidase 4
NAPQI: N-acetyl-p-benzoquinone imineh
ROS: reactive oxygen species
MDA: malondialdehyde
GSH: glutathione
ALT: alanine transaminase
AST: aspartate transaminase
LDH: lactic dehydrogenase
RT-qPCR: reverse transcription quantitative polymerase chain reaction
shRNA: short hairpin RNA
AAV: adeno-associated virus
DMSO: dimethyl sulfoxide
MMP: mitochondrial membrane potential
