# Supplementary material for: GAS1 Promotes Ferroptosis of Liver Cells in Acetaminophen-Induced Acute Liver Failure
Source: Int J Med Sci. 2023 Sep 25;20(12):1616–30. doi: 10.7150/ijms.85114 (PMC10583184; doi:10.7150/ijms.85114)
Supplement: Supplementary file 1 — Supplementary figure. [file ijmsv20p1616s1.zip › Supplementary materials/Figure S1 legend.docx]

**Figure S1** GSH of GAS1^AAV8-OE^ and GAS1^AAV8-vector^ mice liver after 1 hour of APAP administration were assessed.
