# Supplementary figures and images for: GAS1 Promotes Ferroptosis of Liver Cells in Acetaminophen-Induced Acute Liver Failure
Source: Int J Med Sci. 2023 Sep 25;20(12):1616–30. doi: 10.7150/ijms.85114 (PMC10583184; doi:10.7150/ijms.85114)

Figure S1

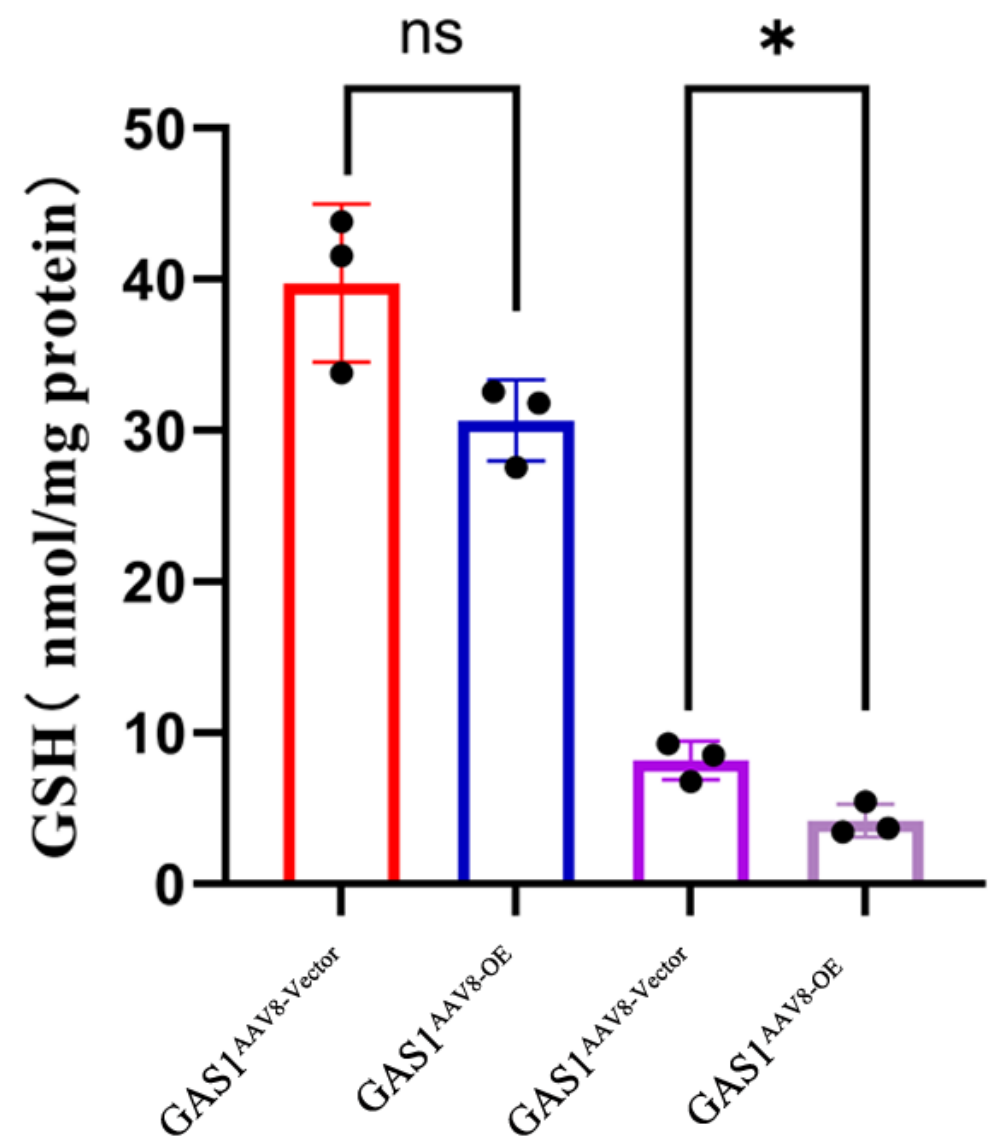

Supplement: Supplementary file 1 — Supplementary figure. [file ijmsv20p1616s1.zip › Supplementary materials/Supplementary figure.pdf]
